# Silanization of SiO_2_ Decorated Carbon Nanosheets from Rice Husk Ash and Its Effect on Workability and Hydration of Cement Grouts

**DOI:** 10.3390/nano11030655

**Published:** 2021-03-08

**Authors:** Ilayda Berktas, Ojas Chaudhari, Ali Nejad Ghafar, Yusuf Menceloglu, Burcu Saner Okan

**Affiliations:** 1 Sabanci University Integrated Manufacturing Technologies Research and Application Center & Composite Technologies Center of Excellence, Teknopark Istanbul, Pendik, 34906 Istanbul, Turkey; iberktas@sabanciuniv.edu (I.B.); yusuf.menceloglu@sabanciuniv.edu (Y.M.); 2 RISE Research Institutes of Sweden, Department Instrastructure and Concrete Construction, Drottning Kristinas väg 26, 114 28 Stockholm, Sweden; ojas.arun.chaudhari@ri.se (O.C.); ali.nejad.ghafar@ri.se (A.N.G.); 3 Faculty of Engineering and Natural Sciences, Sabanci University, Orhanli, Tuzla, 34956 Istanbul, Turkey

**Keywords:** rice husk ash, cementitious grout composite, thermal properties, hydration performance

## Abstract

Rice husk ash (RHA) having a porous sructure and a high amount of amorphous silica nanoparticles (4 nm) decorated on the surface of carbon nanosheets is a suitable and cheap candidate for the use of a grout additive. In this study, neat RHA and functionalized RHA (f-RHA) with three different loadings were successfully incorporated into the cement-bentonite based grouts by adjusting the water to cement ratio. The workability of the developed grouts having RHA-based additives was analyzed in terms of bleeding, density, flow spread, and Marsh cone time. Additionally, the thermal and prolongation of hydration performances of the cementitious grout were enriched by successful attachment of amino-silane functional groups on the RHA surface. The heat of hydration performances of RHA and functionalized RHA introduced cementitious grout composite were assessed by isothermal calorimetry tests, and especially the kinetics of hydration was increased by the addition of RHA. The presence of amino silane groups in f-RHA intensified the heat adsorption by reacting with cement constituents, and thus resulted in the retardation and reduction in the heat flow. Therefore, using an amino-silane coupling agent increased the induction period and hindered the heat of hydration compared to the reference grout. On the other hand, the incorporation of RHA and f-RHA into the cement matrix did not affect the thermal conductivity of the grouts.

## 1. Introduction

In the last decades, global demand for renewable energy technology is growing owing to the high population and economic growth. At this point, geothermal energy can make a great contribution since it is a type of clean renewable energy that generates electricity by adjusting heating and cooling processes by using heat retained in the Earth [1]. Geothermal energy has numerous advantages, unlike other renewable sources such as wind turbines and solar systems, since it does not depend on climatic changes and can be installed in small lands. In shallow geothermal energy systems, thermal conductivity is an important criterion to get high efficiency from the oil surrounding the borehole field. Especially cementitious grout surrounding the borehole plays an essential role in the preservation of the heat flow from the ground to the pipes. However, variation in some of the properties of cementitious grout such as workability, flowability, and thermal conductivity can decrease the performance of the system due to temperature loss during heat transfer. Therefore, many studies have been focused on the performance enhancement of cementitious composites by the incorporation of carbon-based additive materials obtained from virgin and recycled sources [2,3].

Rice husk as raw biomass has also taken great attention of the researchers due to its being one of the major wastes of the world [4], and having a high concentration of silica and organic components and this makes it a suitable source to convert into value-added carbonaceous products [5]. With the proper calcination, rice husk can be recycled into rice husk ash (RHA) which is a highly porous material containing a high amount of amorphous silica and also carries a significant potential for the fabrication of carbon-based nanomaterials. For instance, Wang et al. produced large-scale and controllable graphene quantum dot (GQD) by first synthesizing sp^2^ carbon flakes from rice husks by applying the bottom up technique and then subsequently converting carbon flakes into GQDs through hydrothermal reaction [6]. Moreover, Ismail et al. synthesized graphene from RHA in a cost-effective and easy manner by using potassium hydroxide and applying heat treatment at 800 °C [7]. On the other hand, RHA acting as an additive or an adsorbent or a catalyst carrier can be utilized in various composites structures consisting of rubbery, plastics, and cement constituents, and also in the concrete due to the presence of phases such as SiO_2_, Al_2_O_3_, and others [8,9,10].

RHA contains a significant amount of SiO_2_ (≥90%) that improves the mechanical properties of cement-based mixtures by enhancing the rate of hydration [11,12]. In one of the work, Hamzeh et al. used white rice husk ash (WRHA) as a reinforcing agent in cement composites and showed that water absorption and bulk density of the prepared composites were significantly reduced [13]. Gastaldini et al. focused on the performance of electrical resistivity and compressive strength of concrete mixes by partially replacing RHA with cement and found that a high amount of RHA addition led to the reduction in electrical conductivity and compressive strength of concrete mixes [14,15]. Up to now, the previous studies are mostly on the replacement of RHA with cement to decrease the content of cement used in the grout composition to minimize the environmental impact and provide economic advantage [16]. In addition to the mechanical effect of RHA in the cementitious composites, providing homogeneous dispersion of RHA in the matrix has been considered to optimize the proper grout with the desired properties such as viscosity, flowability, and pumpability. High-quality distribution also affects the water to cement ratio and thus thermal properties of grout mix.

The integration of RHA considerably increases the viscosity of the grout composition, thus affects the flowability and pumpability of the grout negatively. To control these properties, an additional superplasticizer is required to reformulate the grout and get similar workability compared to grout without RHA but this causes an increase in the total cost of operation [15]. Therefore, surface treatment of RHA is needed to overcome high-water demand and adjust the hydrophilicity. Herein, silane coupling agents are widely used as a surface treatment modifier since they can connect organic and inorganic compounds by making bridges with their hydrolyzable and functional groups. Silane coupling agents are highly active chemicals especially under alkaline aqueous conditions which is the general environment in cementitious material such as concrete, mortar, and grout [17]. In one of the studies, Minet et al. intercalated organic groups in calcium silicate hydrate (C–S–H) layers by coprecipitation of organotrialkoxysilanes and tetraethyl orthosilicate (TEOS) mixtures with CaCl_2_ in alkali media [18]. In another work, Franceschini et al. prepared covalently bonded polymer C–S–H composite with the formation of covalent linkages between polymer and C–S–H phases by grafting trialkoxysilane (T-silane) and methyldialkoxysilane (D-silane) to the polymer chains [19]. To sum up, there are some attempts for the development of cement mixes by using rice husk ash as an alternative additive and direct or partial replacing with silica [20,21,22,23] and also most of the published works are mostly focused on the enhancement of strength properties of cementitious composites [24,25]. Besides, the effect of functionalized RHA by silane coupling agents on thermal and mechanical properties of thermoplastic composites was investigated to improve interfacial interactions in the composite structure [26]. On the other hand, there is no study to understand the silanization effect on the interactions between rice husk ash and cement constituents since a direct usage of silanes affects the flowability, strength, and hydration kinetics of cement pastes and mortars [27]. Our study reveals the importance of the chemical interactions between neat and functionalized RHA and bentonite and other cement components such as superplasticizer and different types of silica sands to tailor thermal conductivity and optimize water uptake since water demand is a significant parameter that directly affects the structural and thermal properties of cement composites. It is also known that the adjustment of surface composition of RHA based additives used in the preparation of grout mixes can change the water demand and it is possible to attain high-performance grouts. Furthermore, there is no detailed work for understanding the interfacial interactions of SiO_2_ particles inside the RHA structure with the cement matrix since this type of resource is getting important in terms of sustainability and circular economy.

In our previous studies, thermal conductivity properties of cementitious grout were improved by the incorporation of graphene nanoplatelet/silica and expanded graphite/silica additives in which carbon materials were connected to silica in the presence of silane coupling agents [28,29]. This study focuses on the hydration kinetics and workability of cement paste prepared by the incorporation of 3-aminopropyltriethoxysilane (APTES) treated RHA and also its effect on thermal conductivity. In other words, a different understanding was provided by the utilization of SiO_2_ decorated carbon sheets that were directly obtained by the calcination of rice husk to be used as an additive in cement compositions, and amino functional groups were attached on the surface of RHA to activate silica particles. The covalent attachment of RHA to inorganic C–S–H via APTES can enhance the stability of particles during grout mixing and thus prevent the aggregation by monitoring the rheological properties and fluidity of grouts [30,31]. Comprehensive characterization was carried out the investigations of the chemical composition and structural changes of these functionalized RHA samples. Then, these developed additives were integrated into cement-bentonite based grouts with different loadings, and the effect of amino surface functional groups, and water to cement ratio on workability, and the heat of hydration were studied systematically.

## 2. Materials and Methods

### 2.1. Materials

Rice husk ash with an average particle size of 174 nm and bulk density of 0.1–0.3 g/cc in the form of black powder was collected from Valencia, Spain. 3-Aminopropyl triethoxysilane (APTES, 50825) was purchased by Momentive, Leverkusen, Germany. Acetic acid (100% Anhydrous) was purchased from Isolab, Eschau, Germany. For grout composites, Portland cement: CEM I 42.5 R was obtained from Cimsa, Mersin, Turkey, and Table S1 provides the chemical composition of type I Portland cement used in grout mixes. For aggregates, two types of silica sands (B55 and B20) were selected based on the particle size distribution and purchased from Baskarp Co., Sibelco Nordic AB, Habo, Sweden. Superplasticizer was purchased from Sika, Turkey, and bentonite was obtained from Canbensan Bentonite, Cankiri, Turkey.

### 2.2. Functionalization of Rice Husk Ash by a Silane Coupling Agent

To tailor the surface composition of RHA, silanization was performed to increase the interactions between carbon additive and constituents in grout mixes. Surface functionalization with APTES at a ratio of 1:1 was performed by dispersing 1 g of RHA in 50 mL distilled water via Handheld Ultrasonic Homogenizer from Hielscher Ultrasonics at room temperature. Then, 1 mL APTES as a silane coupling agent was added into the mixture to functionalize the surface of RHA. The pH level of the solution was adjusted to 5.5 by the addition of acetic acid. The solution was poured into the round bottom flask and refluxed overnight at 80 °C. After 24 h, filtration was performed by washing with water twice and dried 24 h in the oven. 

**Figure 1:**
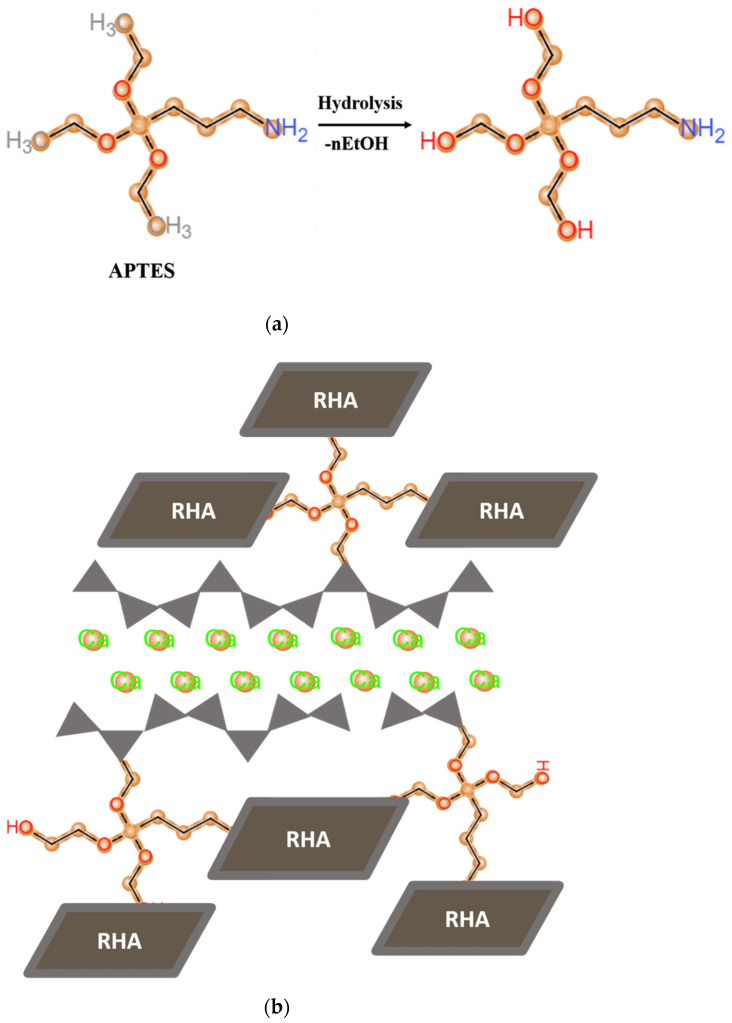
Schematic representations of (**a**) the hydrolysis of APTES and (**b**) the connection of APTES functionalized RHA to the chains of C–S–H via condensation reaction.

As shown in Figure 1, APTES as a silane coupling agent possesses the hydrolysis processes under the alkaline condition and turns into “–OH” hydroxyl groups, which attach at the end and in the middle of calcium silicate hydrate (C–S–H) chains [18]. The remaining organofunctional NH_2_ and hydrolyzed OH groups are then formed covalent bonds with the surface of RHA. 

### 2.3. Cementitious Grout Composition and Mixing Procedure

The cementitious grout samples were prepared by mixing Portland cement, aggregate, superplasticizer, rice husk ash (RHA) or functionalized rice husk ash (f-RHA), and water. The ratio of cement: aggregate was selected as 1:2 by mass to weight. Superplasticizer was added 1% by weight of the cement. Then, RHA or f-RHA was added by 3, 5, and 10 wt% by weight of cement. Table 1 summarizes the composition of cementitious grout with neat RHA and f-RHA.

The cementitious grout samples were mixed by high shear VMA (Reichshof, Germany) mixer with a 60 mm dispersion disk (Dissolver Dispermat^®^ LC, Reichshof, Germany). For mixing, the dry constituents of the grout (cement, RHA, or f-RHA, and sands) were pre-blended in a bag for 1 min. The required quantity of water and superplasticizer, which are shown in Table 1, were added into the mixing bowl, and then a pre-blended grout mixture was added in the mixing bowl. Initially, grout was mixed for 1 min at 2000 rpm, then increased the mixing speed up to 6000 rpm and performed the mixing for 3 min at this stage. The resultant material was cast and cured at 100% relative humidity and 20 °C for 7 days until thermal conductivity testing. Furthermore, the other properties of grouts such as rheology, the density of grout mixes were evaluated using marsh cone test, flow spread testing, and mud balance testing, respectively. In addition, the other properties of grouts such as rheology, the density of grout mixes were evaluated using marsh cone test, flow spread testing, and mud balance testing, respectively. These tests were performed by testing standard SS-EN 445 (SS-EN 445, 2007). The standard mud balance technique was used to measure the density of the grout sample. Further details regarding the preparation and testing for cementitious grout composites can be found in our previous studies [28,29].

### 2.4. Characterization

#### 2.4.1. Characterization of Neat and Functionalized Rice Husk Ash Samples

Thermogravimetric analysis (TGA) was carried out by Mettler Toledo thermal analyzer (TGA/DSC 3+) over the temperature range of 25 °C to 1000 °C at a heating rate of 10 K/min under nitrogen. Raman spectra of neat and functionalized RHA samples were monitored using Renishaw inVia Raman Microscope (Wotton-under-Edge GL12 8JR, United Kingdom) to investigate the structural properties of carbonaceous materials in RHA. The chemical composition of the produced samples was investigated by Thermo Fisher Scientific (Waltham, MA, USA) K-Alpha X-ray Photoelectron Spectroscopy (XPS). Particle size measurement was performed by Anton Paar Litesizer 500. To determine the crystallinity of various RHA samples, X-ray diffraction (XRD) tests were carried out from 2θ = 5° to 80° by Bruker (Billerica, MA, USA) D2 PHASER Desktop with a CuKα radiation source. The morphology and composition of the samples were examined using a Leo Supra 35VP field emission scanning electron microscope (SEM) equipped with an EDS system and JEOL (Tokyo, Japan) JEM-ARM200CFEG UHR- transmission electron microscopy (TEM).

#### 2.4.2. Characterization of the Prepared Grout Mixtures

##### Thermal Conductivity Measurement

Thermal conductivity performance of unmodified and modified RHA included cementitious grout composites were measured by using Hot Disk AB (Göteborg, Sweden) thermal constants analyzer, TPS 2500 S. Specimens with the diameter of 40 mm and the height of 20 mm were molded and cured at 100% relative humidity and 20 °C for evaluation of thermal conductivity.

##### Isothermal Calorimetry Measurement

The heat flow and cumulative heat evolved due to the hydration of each sample were determined under isothermal conditions at 20 ± 1 °C by an eight-channel conduction calorimeter (Tam Air TA Instruments, New Castle, DE, USA) following SS-EN 196-11:2019 [32]. Before mixing, all materials were maintained at 20 ± 1 °C for 24 h. The samples for RHA and f-RHA were prepared by adding 3, 5, and 10 wt% RHA or f-RHA in the neat cement. The mixing was carried out manually in a glass beaker, with the aid of a glass rod at a water to cement ratio of 0.7. Each ampoule was filled with 7 g of paste and the ampoule was instantaneously placed into the isothermal calorimeter channel to measure the heat of hydration at the designated temperature. The entire process, which includes mixing, placement of the paste into the ampoule, and positioning of the ampoule in the calorimeter, took less than 5 min. Data were recorded for a total of 45 h.

## 3. Results and Discussion

### 3.1. Structural, Thermal, and Morphological Properties of Neat and Functionalized Rice Husk Ash

**Figure 2:**
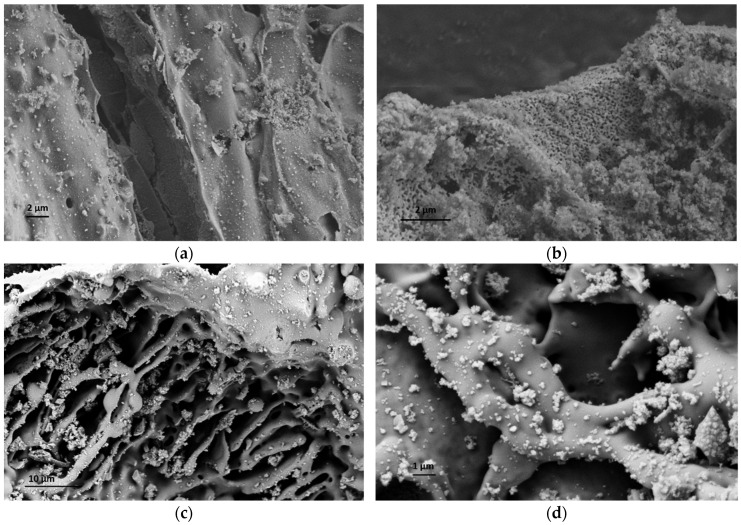
SEM images of (**a**,**b**) RHA, and (**c**,**d**) f-RHA at different magnifications.

**Figure 3:**
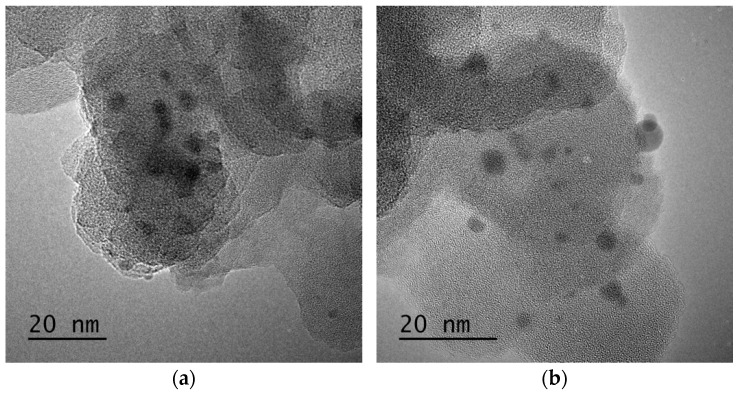
(**a**,**b**) TEM images of neat RHA.

To increase the quality and estimate the potential of RHA in the cement industry, a comprehensive characterization study was carried out to understand the structural properties of RHA, and silanization was applied to change the surface composition of RHA and increase its interactions with the cement components. At the first stage, neat RHA was functionalized by APTES used as a silane coupling agent by following hydrolysis and condensation reactions. To monitor the differences before and after silanization on the morphological properties of RHA, microscopic studies were performed. Figure 2 shows SEM images of neat and functionalized RHA samples at different magnifications. As seen in SEM images, both samples have porous structures, and the presence of silica particles is seen clearly. After functionalization, there was no significant change in the surface properties of RHA but a more porous structure was detected in some regions. Therefore, TEM characterization was conducted for neat RHA to observe silica particle distribution and estimate the particle size. Figure 3 represents TEM images of neat RHA. The results indicated that silica particles with an average particle size of 4 nm were homogeneously placed on the surface of carbon sheets with an average length of 36 nm. In addition to SEM and TEM analysis, EDS characterization was carried out a qualitative analysis of RHA and functionalized RHA to detect the chemical groups available on the surface of the samples. EDS mapping of RHA and f-RHA is shown in Figure S1a,b (provided in supplementary document), and the results clarified the distributions of silica, carbon, oxygen, and other elements on the surface of RHA and f-RHA. Especially EDS of f-RHA contains N peak and thus confirming the attachment of amino groups.

**Figure 4:**
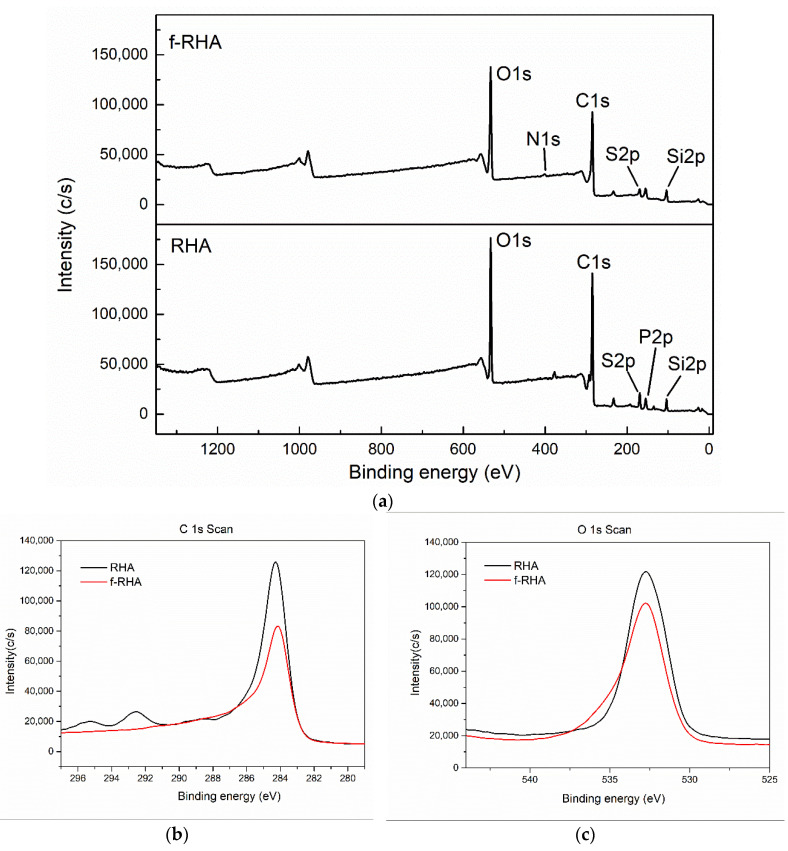
(**a**) XPS survey scan spectra, (**b**) C1s spectra and (**c**) O1s spectra of RHA and f-RHA.

To identify the functional groups of RHA and functionalized RHA samples more specifically, XPS characterization was conducted and C1s, O1s, and N1s signals were measured and the related groups were defined regarding their binding energy of carbon atoms [33]. The XPS peaks of C1s, O1s, and N1s for neat RHA and f-RHA were provided in the XPS survey scan spectra, Figure 4. C1s spectra of RHA and f-RHA in Figure 4b shows a peak at around 284 eV that can be attributed to the C–C bond, while O1s spectra in Figure 4c shows a peak at around 532 eV that can be allocated to the ethoxy group bond O–C [34]. In the case of amine-functionalized RHA, both peaks show a slight decrease in intensity. The C/O ratios of neat RHA and f-RHA were 0.80 and 0.67, respectively.

Table 2 summarizes the XPS results of neat and f-RHA samples. As seen in the table, 1.53 at% of nitrogen was measured in f-RHA due to the attachment of amino groups from APTES. Additionally, the oxygen content was increased from 25.48 to 30.44 at% after functionalization. There is a decrease in carbon content due to an increase in oxygen moiety. On the other hand, silicon amount increased from 5.88 to 8.15 at% and this increase in silicon amount was expected due to the attachment of silane groups during functionalization. Therefore, XPS analysis confirmed the successful incorporation of amino groups on the surface of RHA. The similar result which was observed in the EDS analysis also verified the confirmation of XPS analysis.

**Figure 5:**
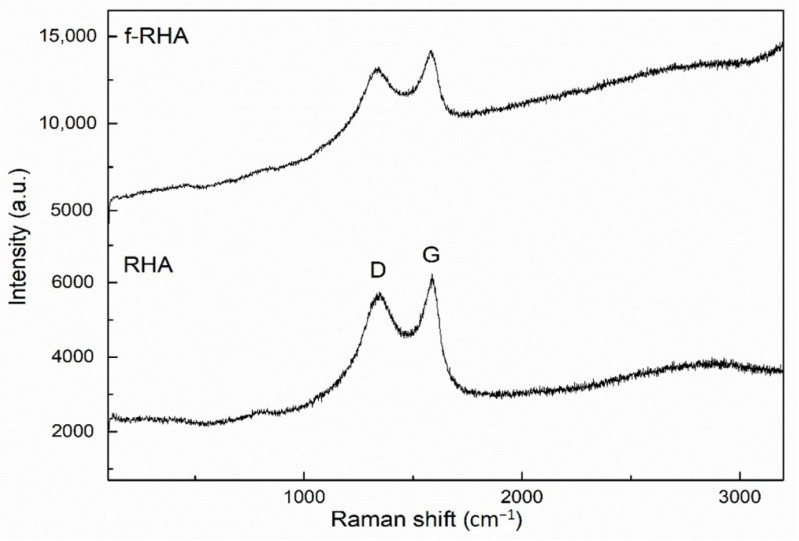
Raman spectra of RHA and f-RHA.

To examine the structural changes in functionalized RHA, Raman spectroscopy was used to investigate the properties of carbonaceous structure in RHA, and Raman spectra of the samples are given in Figure 5. There are two main peaks that appeared at around 1343 and 1586 cm^−1^ attributed to D and G peaks, respectively. D peak attributes the degree of defects in the structure while G peak indicates the vibrational mode of sp^2^ in carbonaceous materials [28]. The structural changes were compared by taking into consideration the intensity ratios of the D and G bands (I_D_/I_G_). As shown in Table S2, I_D_/I_G_ values of neat and f-RHA were calculated as 0.92 and 0.93, respectively. This change stems from an increase in surface amino functional groups due to chemical treatment and thus the successful functionalization is verified by structural characterization. 

**Figure 6:**
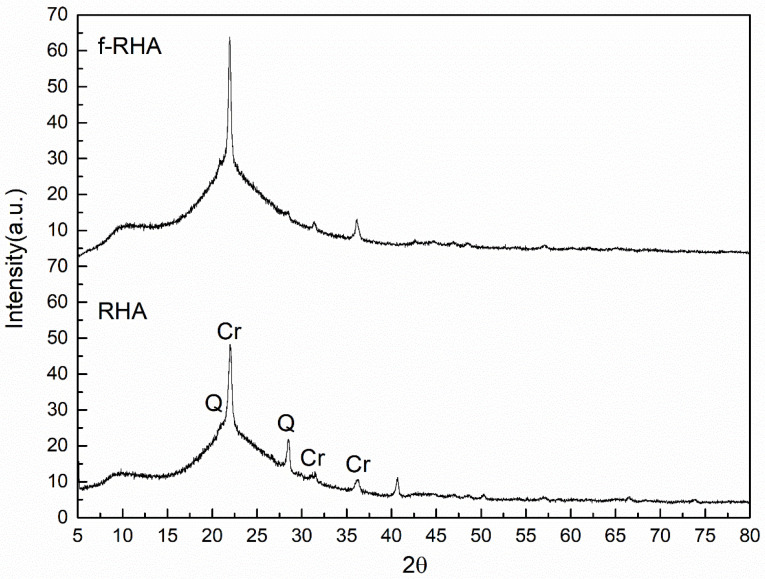
XRD patterns of RHA and f-RHA (Cr and Q signs in the XRD spectra of RHA represent cristobalite and Quartz peaks, respectively [35,36,37]).

Figure 6 shows XRD patterns of RHA and functionalized RHA. Unmodified RHA shows high crystalline cristobalite (Cr) peak at 2θ = 22° hkl (004) and 2θ = 36.3° hkl (040) and a small Quartz’s (Q) peak at 2θ = 21.1° hkl (220) [15]. Additionally, there is a broad peak between 2θ = 20–30° overlapped with Cr peak indicating the presence of carbon and silica in the structure. After the modification by APTES, the peak at around 2θ = 28.62° belonging to Q was disappeared and the intensity of Cr peak increased in the XRD pattern of f-RHA and thus crystallinity of RHA was slightly increased.

**Figure 7:**
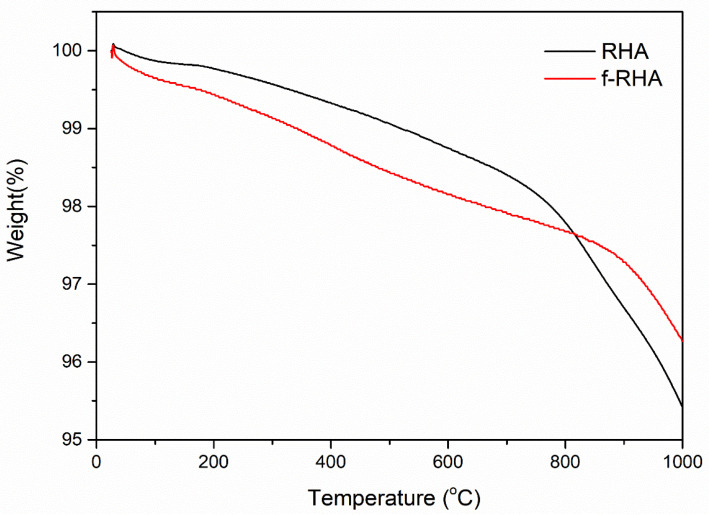
TGA curves of neat RHA and f-RHA samples.

Besides, thermal degradation behaviors of neat and functionalized RHA samples were investigated by TGA. Figure 7 depicts neat RHA and f-RHA samples. Neat RHA showed higher thermal stability until 1000 °C and there was only 4.58% weight loss in the structure. After functionalization, the weight loss is around 3.73%. There is a slight difference in the degradation curves of neat and functionalized RHA samples. To conclude, the surface of RHA was treated by silane-based agents successfully and the presence of functional groups was confirmed by spectroscopic and gravimetric techniques.

### 3.2. The Characteristic Properties of Grout Mixtures

After the preparation of f-RHA samples, grouts were formulated using neat RHA and f-RHA at the dosage of 3, 5, and 10 wt%. Marsh cone flow time, mini-slump flow diameter, density, bleeding of grout samples, and the thermal conductivity results are shown in Table 3. The properties of grouts were characterized by comparing workability, thermal conductivity, and heat of hydration results.

#### 3.2.1. The Workability Properties of Grout Mixtures

The workability properties such as marsh cone time, bleeding, and flow spread were maintained in the benchmark range by adjusting water to cement ratio. It was found that f-RHA grout samples require less water to achieve similar workability to RHA grout samples. This comes from the functionalization with APTES leading to the enhancement of the wettability of RHA by water and thus an increase in the workability of the grout [38].

**Figure 8:**
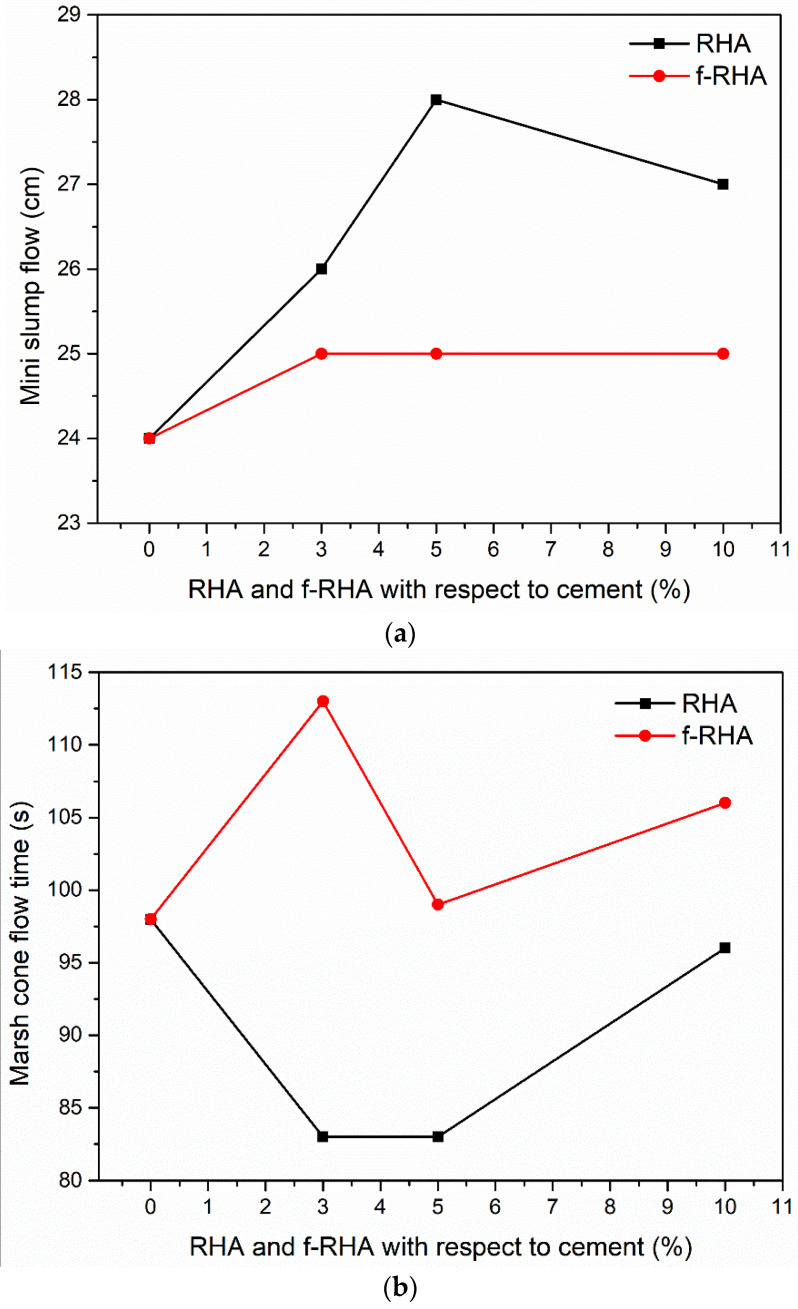
(**a**) Mini slum flow vs RHA and f-RHA amounts with respect to cement curves, and (**b**) Marsh cone flow time vs RHA and f-RHA amounts with respect to cement curves.

Deformability and fluidity (workability properties) of the grout mixtures are examined and Figure 8 represents the curves of Mini slum flow and marsh cone flow as a function of RHA amount. According to Figure 8a, for the moderate addition of RHA (3 and 5 wt%), slump flow was increased with the amount of RHA in the grout. However, slump flow was reduced at 10 wt% RHA addition. On the other hand, for f-RHA, slump flow stayed constant at all the dosages. Similar behavior was monitored in the marsh cone testing. As shown in Figure 8b, for the moderate addition of RHA, marsh cone flow time was reduced and at the addition of 10 wt% RHA, marsh cone time was increased. Moreover, at the lower additions (3 and 5 wt%), marsh cone time for RHA grout is lower when compared to f-RHA grout samples, but at higher additions (10 wt%), both samples showed a rise in the marsh cone time. These results showed that at the addition of 10 wt%, both RHA and f-RHA have impacted the viscosity and yield point of the grout in a similar magnitude. Including a high percentage of RHA or f-RHA has reduced the fluidity of grout samples due to the high-water absorption capacity of RHA and f-RHA compared to Portland cement. As it was observed from SEM images, the porous structures of RHA and f-RHA having voids and channels can cause more water uptake [39]. In addition, the density and total weight of cementitious materials were kept constant, and then the volume of the grout mixture was increased by the incorporation of RHA. An increase in the volume of paste leads to an increase in plasticity and cohesiveness of grout mix that reduces the overall fluidity of grout mix [40]. Therefore, high water to cement ratio was required to optimize the grout workability in the acceptable range. 

The density values of each grout sample at different additions for RHA and f-RHA are given in Table 3. The density of grout samples was slightly decreased as RHA or f-RHA content increased in the grout mix. Both RHA and f-RHA have no significant effect on the wet density of cementitious grout. Finally, the dimensional stability of grout mixes is described by bleeding test, in which shrinkage of grout after 24 h is evaluated. It was found that all samples showed negligible shrinkage according to the defined technical specs provided in benchmarking study (<2%). Furthermore, f-RHA grout showed less shrinkage in comparison to RHA grout samples, which can be attributed to the high water retention capacity of f-RHA due to the presence of amino-silane functional groups [38]. 

#### 3.2.2. Thermal Conductivity of Rice Husk Ash-Based Grouts 

**Figure 9:**
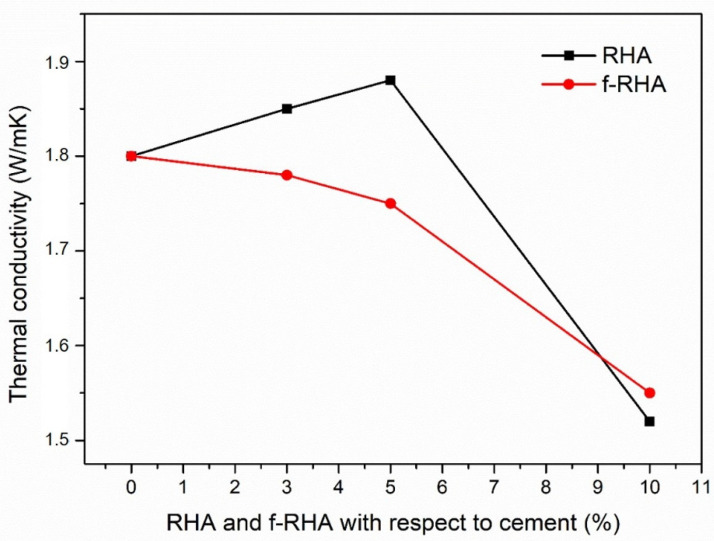
Thermal conductivity of grout containing RHA and f-RHA samples at different loadings.

The thermal conductivity of the grout depends on the additives, which are bentonite, silica sands, and superplasticizer, as much as it depends on the water uptake. Bentonite has a relatively low thermal conductivity that typically ranges from 0.65 to 0.90 W/mK under the saturated condition [41]. Since the low thermal conductivity of bentonite might reduce the overall thermal conductivity of the reference grout, the addition of an excessed amount of bentonite was avoided during grout mixing. On the other hand, superplasticizer does not have a significant influence on the thermal conductivity due to its small proportion in the grout. To evaluate the influence of RHA and f-RHA on the thermal conductivity, the amounts of bentonite and superplasticizer were kept the same in the grout formulations. Overall, the thermal conductivity of reference grout was measured as 1.80 W/mK. Furthermore, the thermal conductivity results of grout composites containing RHA and f-RHA with three different dosages are presented in Figure 9.

After the integration of 3 and 5 wt% RHA, there was a slight increase in thermal conductivity. However, as the RHA amount reached 10 wt%, thermal conductivity has started to decrease. This showed that 5% is the optimum loading of RHA to achieve maximum thermal conductivity of the grout. On the other hand, there was a decline in the thermal conductivity of grouts containing f-RHA. At the loading of 10 wt% f-RHA, a sharp loss in thermal conductivity was detected which was consistent with 10 wt% RHA based grout. This also supports that there is a direct relation between thermal conductivity and the amount of water uptake in the grout [28,29]. Furthermore, f-RHA becomes more hydrophilic after the functionalization by APTES and retains more water during the hydration of grout. In other words, since available water content has an adverse effect on the thermal conductivity, the integration of f-RHA, which has a tendency to retain the water in the grout, leads to lower thermal conductivity [42]. In the case of neat RHA, extra water was required during grout mixing which was retained during hydration of grout. Furthermore, the thermal conductivity can be reduced due to accessible porosities and lower density microstructure of grout caused by the addition of RHA or f-RHA [43]. Consequently, both neat RHA and f-RHA did not cause a significant improvement in thermal conductivity of bentonite-cement based grouts.

#### 3.2.3. The Effects of RHA on the Heat of Hydration 

**Figure 10:**
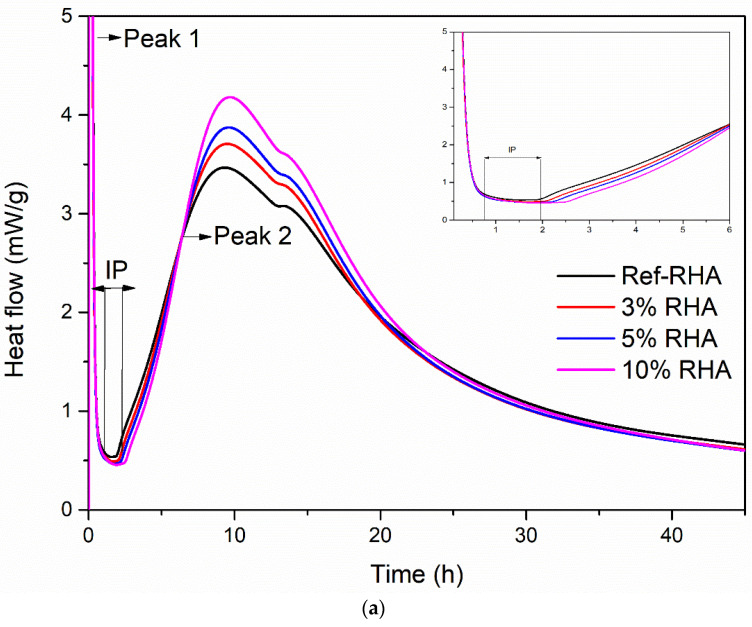

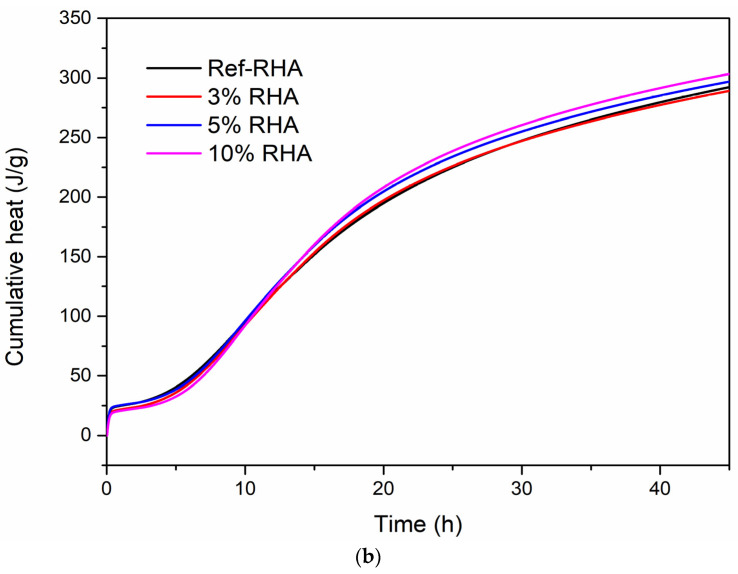
Hydration heat curves of RHA based grouts: (**a**) heat flow and (**b**) cumulative heat release.

**Figure 11:**
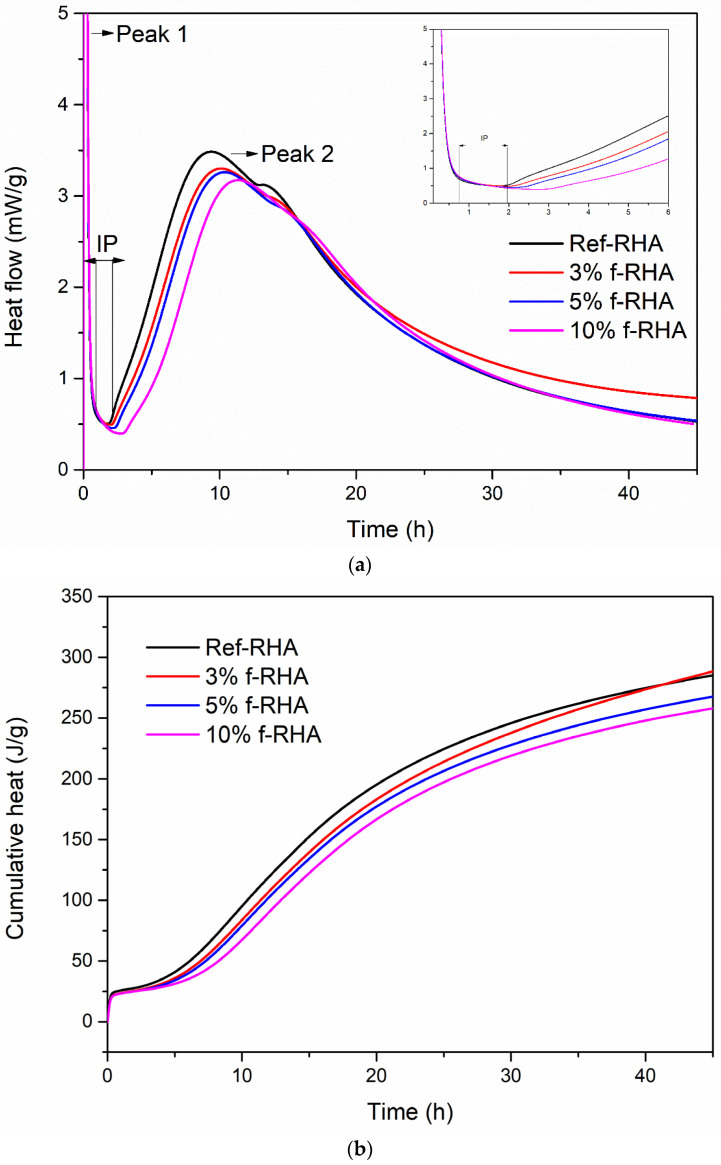
Hydration heat curves of f-RHA based grouts: (**a**) heat flow and (**b**) cumulative heat release.

When water is mixed with the constituents of grouts, an exothermic reaction occurs between cement and water, and thus resulting in heat release [44]. The reaction proceeds in the different phases and depends on the cement type, composition, water to cement (w/c) ratio, and additives, the magnitude of heat release varies as a function of time. Measurement of the rate of heat evolution provides an understanding of the rate of hydration of grout in the presence of RHA and f-RHA. The hydration heat release of each grout sample is estimated by using an isothermal conduction calorimeter. To examine the effect of only RHA and f-RHA, other workability enhancing additives (superplasticizer and bentonite) were excluded from grout mix, and water to cement ratio (w/c = 0.7) was considered as an adjustable parameter. Figure 10 and Figure 11 represent the heat flow and cumulative heat release curves of paste containing cement with RHA and f-RHA, respectively. In the heat flow curves (Figure 10a and Figure 11a), enlarged plots were displayed in the first 6 h of hydration in detail. The heat flow and cumulative heat release for each sample were normalized by using weight of cement. In heat flow curves, the first heat flow peak (Peak 1) is due to cement wetting (which was out of the scale in the graph), shows an induction period of low heat flow and an acceleration period and then the second peak called Peak 2 associates with the hydration of Ca_3_SiO_5_ (C_3_S) [33]. In addition, the weak shoulder of Peak 2 is directly related to sulfate depletion and the reaction of Ca_3_Al_2_O_6_ (C_3_A) for the formation of ettringite due to the nucleation [45,46]. 

The calorimetric results indicated that the use of neat RHA in cement increased the kinetics of hydration compared to the reference paste, as seen in Figure 10a. In addition, the kinetics of hydration was enhanced by increase RHA content. Moreover, the heat flow peak values for the samples having 3, 5, and 10 wt% were 3.71, 3.87, and 4.18 mW/g, respectively. Herein, the porous structure of RHA leads to the absorption of more water during the grout mixing [47]. The absorbed water in RHA has reduced the degree of hydration of the cement at an early stage as less water is available to react with the cement particles resulting in the prolonged induction period, as shown in Figure 10a. However, the absorbed water in the RHA structure can act as an additional water reservoir during the hydration process. As the hydration proceeded, initially captured water in cement hydration reaction reduced the stoichiometric amount of water to keep the rate of hydration constant, and thus the water captured in the pores of RHA was released. This additional water can accelerate the hydration of cement at later stages [48]. As seen in Figure 10b, cumulative heat released during the hydration process is enhanced by the addition of RHA and this trend is similar to the heat flow analysis in Figure 10a.

Figure 11a exhibits the heat flow evaluation of paste containing 3, 5, and 10 wt% f-RHA compared to the reference (0 wt% RHA). After APTES functionalization, the heat flow values for the grouts containing 3, 5, and 10 wt% were measured as 3.29, 3.25, and 3.16 mW/g, respectively. The addition of f-RHA in paste was altered the hydration curve significantly, extended the induction period, and reduced the magnitude of the second peak. The incorporation of 3 and 5 wt% f-RHA was increased the induction period as 15 and 30-min, respectively, whereas 10 wt% f-RHA increased the induction period by 60 min in comparison to the reference grout. Furthermore, the cumulative heat evolution in the paste was significantly reduced by the addition of f-RHA (Figure 11b). The prolonged induction period and reduction in heat flow can be attributed to the influence of silane-based functional groups on the surface of f-RHA. The silane groups tend to adsorb and react with the surfaces of components in the cement and thus this can hinder the cement hydration reaction. As the amount of f-RHA increases, APTES in f-RHA might further intensify the adsorption and result in the retardation and reduction in the heat flow [17].

## 4. Conclusions

In the present study, a different approach was brought about the utilization of biomass of rice husk and its conversion into an additive in cement composites to monitor the changes in thermal conductivity and grout properties. Therefore, a detailed characterization was carried out to understand the physical, chemical, thermal, and morphological characteristics of RHA by using spectroscopic and microscopic techniques. To increase the interfacial interactions of RHA with the constituents of cement such as bentonite, silica sand, and superplasticizers, APTES functionalization was applied to the surface of RHA by hydrolysis process to form covalent bonds by taking into consideration our previous studies conducted by using expanded graphite and graphene nanoplatelet. The surface chemical composites confirmed an increase in nitrogen content meaning the attachment of amino functional groups. Then, grout formulation was developed successfully by getting ideal water to cement ratio and RHA based additive amount. Benchmarking studies and experimental properties of grouts containing RHA and f-RHA showed that there was a slight increase in thermal conductivity of RHA based grout up to 5 wt% loading, and then at higher additive loadings, thermal conductivity was decreased. According to workability analysis, f-RHA grout samples got less water to obtain similar workability of RHA based grout since the functionalization with APTES leading to the enhancement of the wettability of RHA by water increased the workability of the grout. In addition, APTES functionalized RHA showed the tendency to adsorb and react with the surfaces of cement components and thus resulting in inhibiting the cement hydration reaction and increasing the induction time regarding the calorimetry analysis.

This study indicated that surface treatment by APTES as a silane coupling agent can easily activate RHA, and as the hydrophilicity of the additive surface affects the viscosity of grout directly by altering marsh cone time. Additionally, the thermal conductivity of grouts has started to decrease slightly by increasing the additive amount of both RHA and functionalized RHA due to an increase in water uptake. To conclude, this study shows the potential of RHA as an additive which is a highly desirable and cheap source for cement owing to its high concentration of pure amorphous silica and the presence of nanometer-sized carbon sheets.

## Figures and Tables

**Table 1:** Composition of cement-bentonite based grouts with neat RHA and f-RHA.

Sample Name	Control	Neat RHA			f-RHA		
	C0	RHA3	RHA5	RHA10	f-RHA3	f-RHA5	f-RHA10
RHA (wt%)	0	3	5	10	3	5	10
Cement (g)	930	930	930	930	930	930	930
Silica Sand 1 (g) B20	900	900	900	900	900	900	900
Silica Sand 2 (g) B55	900	900	900	900	900	900	900
Bentonite (g)	10	10	10	10	10	10	10
RHA (g)	-	27.9	46.5	93	-	-	-
f-RHA (g)	-	-	-	-	27.9	46.5	93
Superplasticizer (g)	18.6	18.6	18.6	18.6	18.6	18.6	18.6
Water (g)	650	680	700	730	670	690	720

**Table 2:** XPS results of RHA and f-RHA samples in terms of atomic percentages.

Sample Name	Carbon (at%)	Oxygen (at%)	Silicon (at%)	Nitrogen (at%)	Sulphur (at%)	Others (at%)
RHA	62.81	25.48	5.88	-	3.52	2.31
f-RHA	57.46	30.44	8.15	1.53	2.42	-

**Table 3:** Benchmark and experimental properties of grouts containing RHA and f-RHA.

Sample Name	Benchmark	Control	Neat RHA			f-RHA		
		C0	RHA3	RHA5	RHA10	f-RHA3	f-RHA5	f-RHA10
Rice husk ash (wt%)		0	3	5	10	3	5	10
Water to cement ratio		0.70	0.73	0.75	0.78	0.72	0.74	0.77
Marsh cone time (s)	100–120	98	83	83	96	113	99	106
Flow spread (cm)	23–28 cm	24	26	28	27	25	25	25
Bleeding (%)	<2%	0.5	0.6	0.6	0.6	0.3	0.3	0.2
Density (g/cc)	>1.3	2.10	2.09	2.07	2.05	2.11	2.08	2.04
Thermal conductivity (W/mK)		1.80	1.85	1.88	1.52	1.78	1.75	1.55

## Data Availability

The data presented in this study are available on request from the corresponding author.

## Supplementary Materials

The following are available online at https://www.mdpi.com/2079-4991/11/3/655/s1, Figure S1. SEM image, EDS analysis, and Elemental mapping analysis of (a) RHA and (b) f-RHA, Table S1. The properties of ordinary type I Portland cement, Table S2. Summary of Raman peak intensities and I_D_/I_G_ ratios of RHA and f-RHA.
