# Supplementary material for: Silanization of SiO2 Decorated Carbon Nanosheets from Rice Husk Ash and Its Effect on Workability and Hydration of Cement Grouts
Source: Nanomaterials (Basel). 2021 Mar 8;11(3):655. doi: 10.3390/nano11030655 (PMC7999737; doi:10.3390/nano11030655)
Supplement: Supplementary file 1 [file nanomaterials-11-00655-s001.pdf]

nanomaterials

Article

Silanization of SiO_2 Decorated Carbon Nanosheets from Rice Husk Ash and Its Effect on Workability and Hydration of Cement Grouts

Ilayda Berktas [1], Ojas Chaudhari [2], Ali Nejad Ghafar [2], Yusuf Menceloglu [1,3] and Burcu Saner Okan [1,*]

[1] Sabanci University Integrated Manufacturing Technologies Research and Application Center & Composite Technologies Center of Excellence, Teknopark Istanbul, Pendik, 34906 Istanbul, Turkey; iberktas@sabanciuniv.edu (I.B.); yusuf.menceloglu@sabanciuniv.edu (Y.M.)
[2] RISE Research Institutes of Sweden, Department Instrastructure and Concrete Construction, Drottning Kristinas väg 26, 114 28 Stockholm, Sweden; ojas.arun.chaudhari@ri.se (O.C.); ali.nejad.ghafar@ri.se (A.N.G.)
[3] Faculty of Engineering and Natural Sciences, Sabanci University, Orhanli, Tuzla, 34956 Istanbul, Turkey
* Correspondence: burcu.saner@sabanciuniv.edu, Tel.: +90-216-300-13-06

Abstract: Rice husk ash (RHA) having a porous sructure and a high amount of amorphous silica nanoparticles (4 nm) decorated on the surface of carbon nanosheets is a suitable and cheap candidate for the use of a grout additive. In this study, neat RHA and functionalized RHA (f-RHA) with three different loadings were successfully incorporated into the cement-bentonite based grouts by adjusting the water to cement ratio. The workability of the developed grouts having RHA-based additives was analyzed in terms of bleeding, density, flow spread, and Marsh cone time. Additionally, the thermal and prolongation of hydration performances of the cementitious grout were enriched by successful attachment of amino-silane functional groups on the RHA surface. The heat of hydration performances of RHA and functionalized RHA introduced cementitious grout composite were assessed by isothermal calorimetry tests, and especially the kinetics of hydration was increased by the addition of RHA. The presence of amino silane groups in f-RHA intensified the heat adsorption by reacting with cement constituents, and thus resulted in the retardation and reduction in the heat flow. Therefore, using an amino-silane coupling agent increased the induction period and hindered the heat of hydration compared to the reference grout. On the other hand, the incorporation of RHA and f-RHA into the cement matrix did not affect the thermal conductivity of the grouts.

Keywords: rice husk ash; cementitious grout composite; thermal properties; hydration performance

Citation: Berktas, I.; Chaudhari, O.; Ghafar, A.N.; Menceloglu, Y.; Okan, B.S. Silanization of SiO_2 Decorated Carbon Nanosheets from Rice Husk Ash and Its Effect on Workability and Hydration of Cement Grouts. *Nanomaterials* **2021**, *11*, 655. https://doi.org/10.3390/nano11030655

Academic Editor: Alexander Kromka

Received: 4 January 2021
Accepted: 8 February 2021
Published: 8 March 2021

Publisher's Note: MDPI stays neutral with regard to jurisdictional claims in published maps and institutional affiliations.

Copyright: © 2021 by the authors. Licensee MDPI, Basel, Switzerland. This article is an open access article distributed under the terms and conditions of the Creative Commons Attribution (CC BY) license (http://creativecommons.org/licenses/by/4.0/).

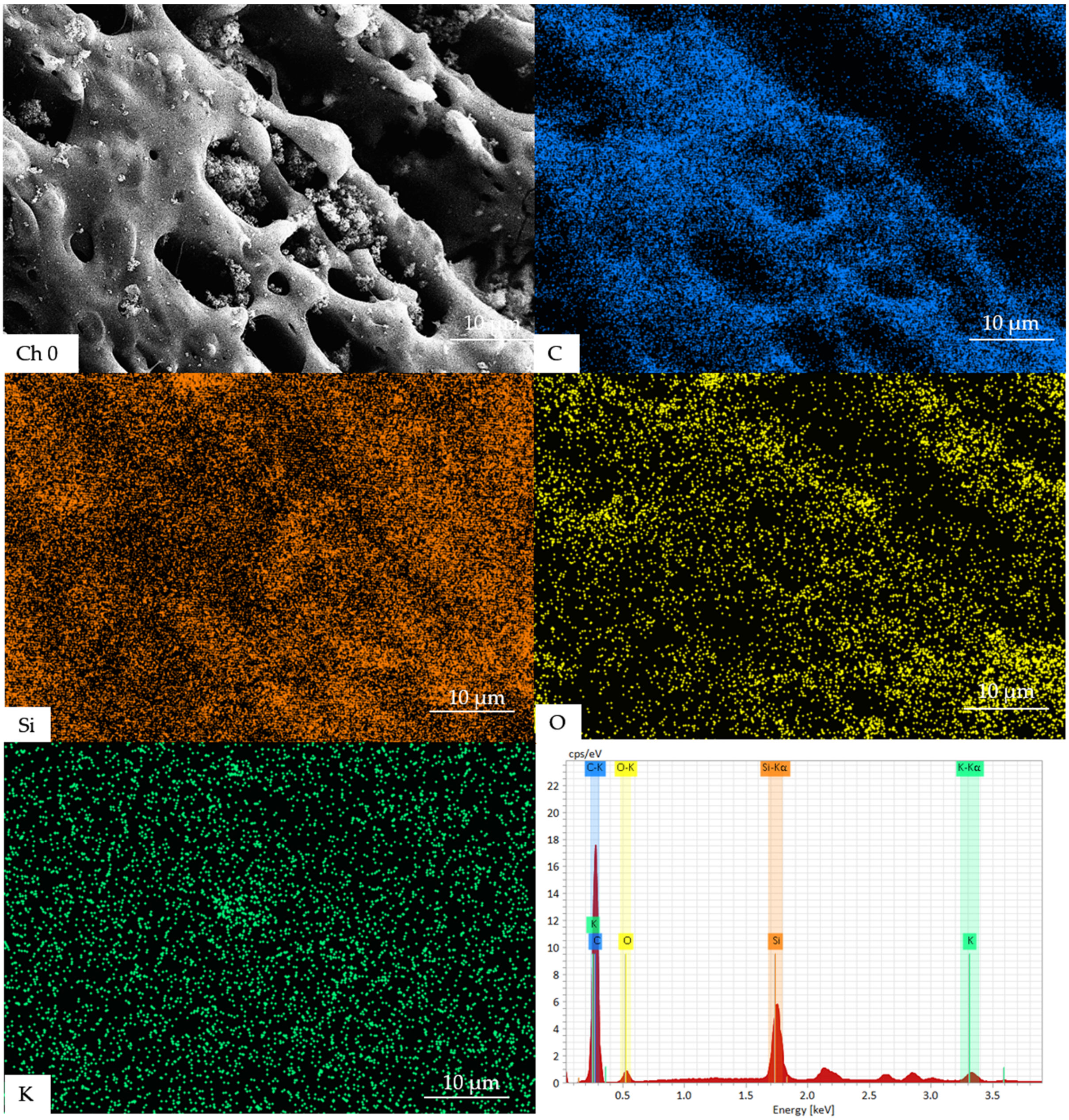
Ch 0
10 µm
C
10 µm
Si
10 µm
O
10 µm
K
10 µm
cps/eV
C-K
O-K
Si-Kα
K-Kα
Energy [keV]

(**a**)

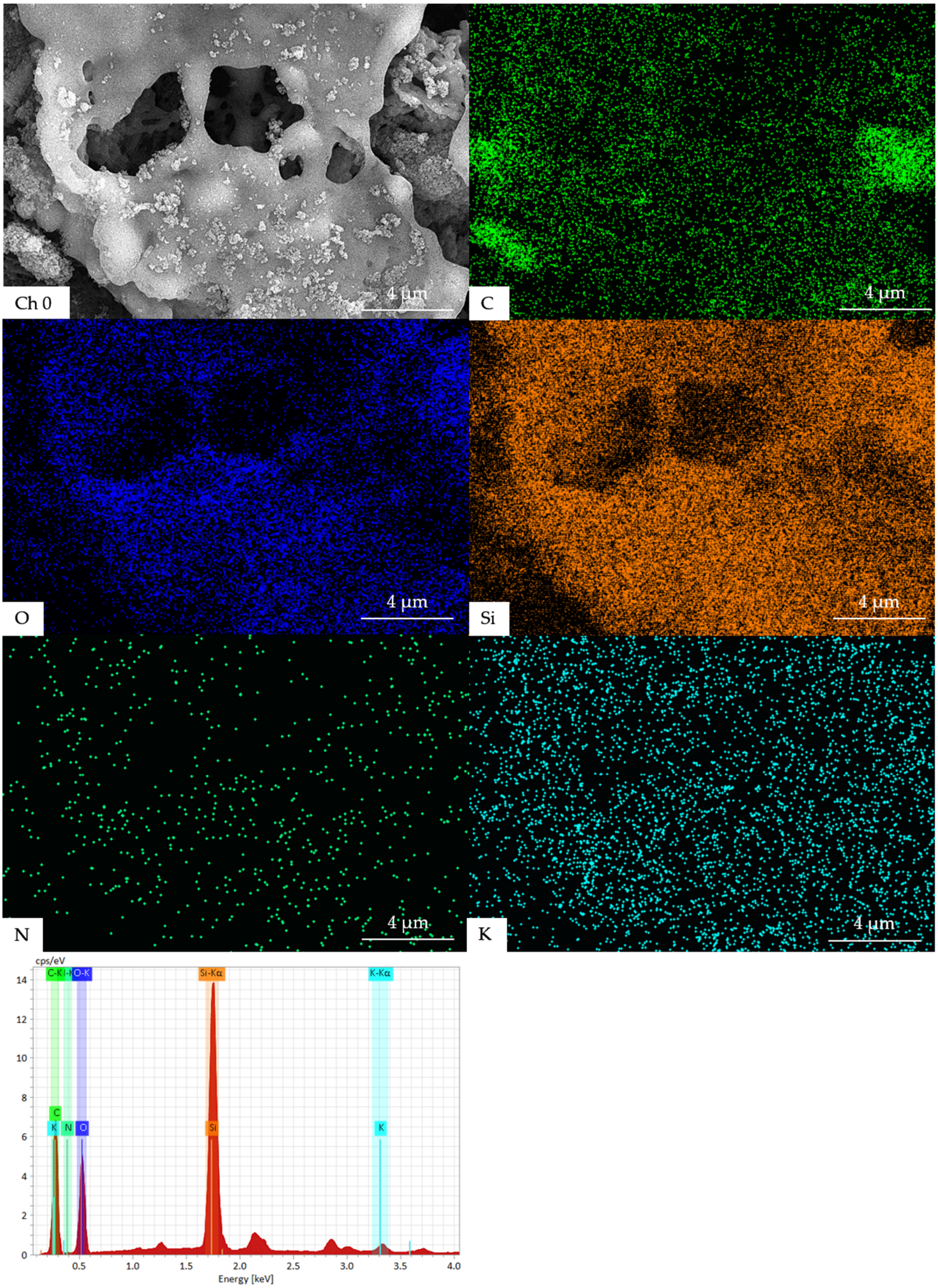

(**b**)

Figure S1. SEM image, EDS analysis, and Elemental mapping analysis of (**a**) RHA and (**b**) f-RHA.

Table S1. The properties of ordinary type I Portland cement.

**Cement Type**	**CEM I 42.5**
**Provider**	CIMSA, Turkey
**Chemical Composition**	Assay (wt%)
**SiO_2**	19.55
**Al_2O_3**	4.48
**Fe_2O_3**	2.92
**CaO**	63.54
**MgO**	2.41
**SO_3**	3.34
**Na_2O**	0.34
**K_2O**	0.86
**TiO_2**	0.28
**Cement Mineral Composition**	Assay (wt%)
**C_3S**	69.37
**C_2S**	8.76
**C_3A**	3.78
**C_4AF**	9.67

Table S2. Summary of Raman peak intensities and I_D/I_G ratios of RHA and f-RHA.

**Samples**	**D intensity (a.u.)**	**G intensity (a.u.)**	**I_D/I_G**
RHA	5746.9	6233.9	0.92
f-RHA	13,273.4	14,206.3	0.93
